# PROfessionalism in Partnership for Education Research (PROPER) study: a novel online initiative approach to professionalism education

**DOI:** 10.1186/s12909-026-08908-2

**Published:** 2026-04-29

**Authors:** Asil Sadeq, Shaista S. Guraya, G. P. Kearney, A. Ryan, E. Clarke, M. Smith, A. Bensaaud, S. Hand, Salman Y. Guraya, F. Doyle, F. Boland, M. T. Harbinson, D. W. Harkin

**Affiliations:** 1 Centre for Professionalism in Medicine and Health Sciences, Faculty of Medicine and Health Sciences, RCSI University of Medicine and Health Sciences, Dublin, Ireland; 2 Institute of Learning, Mohammed Bin Rashid University of Medicine and Health Sciences, Dubai Health, Dubai, United Arab Emirates; 3 School of Medicine, Dentistry and Biomedical Sciences, Queens University Belfast, Belfast, UK; 4 College of Medicine, Gulf Medical University, Ajman, UAE; 5 Department of Health Psychology, School of Population Health, RCSI University of Medicine and Health Sciences, Dublin, Ireland; 6 Data Science Centre, School of Population Health, RCSI University of Medicine and Health Sciences, Dublin, Ireland

**Keywords:** Medical professionalism, Educational interventions, E-learning, Theory of planned behaviour, Professional behaviour, Mixed methods, Cultural sensitivity, Undergraduate students

## Abstract

**Introduction:**

Medical education must ensure medical students graduate with the appropriate values, attitudes, and behaviours that allow them to work collaboratively with patients and families from a range of diverse cultural backgrounds. The PROfessionalism in Partnership for Education Research (PROPER) study aimed to assess the impact of an online educational intervention on medical professionalism for undergraduate medical students in two jurisdictions, focusing on behaviours and cultural sensitivity.

**Methods:**

The PROPER study employed a quasi-experimental, mixed-methods design with intervention and control groups formed by participants self-selection. underpinned by the Theory of Planned Behaviour (TPB), the intervention comprised four online workshops, using scenario analyses and reflective practices, addressing confidentiality, raising concerns, self-care, and cultural awareness. Quantitative survey data were collected at three time points: pre-workshop, immediate post workshop (short-term) and three months follow-up and qualitative data were obtained through focus group discussions (FGDs). Quantitative analyses included paired and independent t-tests, while qualitative data were analysed thematically.

**Results:**

Forty-one participants (intervention = 31, control = 10) were included. The most notable change was observed in perceived behaviour control for self-care, which significantly improved from pre- to post-workshop (*P* < 0.05). For other constructs, improvements were observed but did not reach statistical significance. Other TPB items showed similar directions of change, where decreases or no improvements were observed. FGDs (*n* = 5, 24 participants) analysis identified four key themes across the combined workshops: perspective enhancement, enabling self, dialogic reflection, and strengthening axiology. These themes highlighted evidence in increased self-awareness, confidence, and the early development of professional values among participants.

**Discussion:**

Despite limited statistical evidence, qualitative insights suggest PROPER intervention may be a promising model for online undergraduate professionalism education, combining a structured programme and reflection to enhance collaboration and cultural competence. Challenges such hierarchical norms, structural barriers and participants’ relative lack of clinical experience were perceived to limit long-term behaviour change. More evidence is needed in this area, focusing on incorporating experiential learning and addressing contextual barriers at a larger sample size to sustain professionalism behaviour.

**Supplementary Information:**

The online version contains supplementary material available at 10.1186/s12909-026-08908-2.

## Background

Doctors work in increasingly diverse environments, encountering professionally challenging situations [1]. Students also have professional obligations like qualified doctors during clinical learning, underscoring the importance of early professionalism teaching [1–3]. Unprofessional behaviour in medical school is associated with subsequent disciplinary action by medical regulatory boards during clinical practice, further justifying this focus [4]. Therefore, medical educators must prepare students with the values, attitudes, and behaviours needed to navigate professionalism dilemmas and collaborate with culturally diverse patients and families [2, 3, 5]. The mission for medical education is to ensure patients will receive the same degree of medical professionalism (MP) practice irrespective of where their doctor graduated. MP is a vital competency for patient safety and care quality, forming a core competency in undergraduate medical students [6]. Medical institutions incorporate professionalism teaching into formal curricula typically through diverse approaches; combination of longitudinal strands, stand-alone modules, and clinical role modeling opportunities, reflecting a broad consensus that professionalism is a core competency however requiring structured development rather than incidental. Despite this widespread, challenges remain in aligning taught values with the realities of clinical practice; where students frequently encounter behaviours, norms, and pressures that contradict formal instruction where the hidden curriculum can convey contradictory message [7]. This tension between the formal and hidden curricula can dilute the impact of MP teaching, creating inconsistencies that influence students’ ability to internalise and sustain professional behaviour promoted in educational settings [8–10].

Research has investigated strategies to minimize the effects of the hidden curriculum using reflective practice, support groups, guided experimental workshops and scenario-based analysis, reflecting the diverse pedagogical strategies used to support professional behaviour (PB) and professional identity formation within MP education [9, 11]. This underscores the need to target values, behavioural intentions, self-efficacy, and observable behaviours [10, 12]. Evidence on theory-based interventions, validated tools and long-term sustainability with focus on cultural sensitivity to mitigate challenges of hidden curriculum remains limited, however research suggest that supporting PB in medical education is measurable, requires embedding a cognitive foundation, social learning in the curriculum and involves students in the active construction of their identities [5].

Similar gaps between research, education and practice are particularly relevant on the island of Ireland, which includes the separate political jurisdictions of Northern Ireland, United Kingdom (UK) and the Republic of Ireland; these have different medical-education and regulatory frameworks, and yet share medical training, people and geography. This creates an additional challenge and provides a unique opportunity for studying the benefits of shared online MP education. Thus, PROfessionalism in Partnership for Education Research (PROPER) study addresses this by developing a best-practice model for MP education and PB development across borders. With the evidence suggesting that PBs are often formally assessed [8, 13–15] and using online curated workshops with scenario analysis, PROPER study aims to (i) co-educate medical students from two institutions in two regions; and (ii) assess the potential impact of an online educational intervention on MP for undergraduate medical students in the two jurisdictions, focusing on behavioural change and hidden curriculum sensitisation.

## Methods

### Design

PROPER is a quasi-intervention mixed-methods design, designed in accordance with the Consolidated Standards of Reporting Trials (CONSORT) statement [16]. Underpinned by social cognitive theory and the theory of planned behaviour (TPB) as detailed in a prior publication [17], PROPER study comprises three components:

1. Workshops and scenario analysis: Four online workshops on Microsoft (MS) Teams^®^ on key professionalism competencies.
2. TPB-based surveys: measured at three time points: pre-workshop, post-workshop, and three-month follow-up, with a comparator group.
3. TPB-based focus group discussion (FGD): semi-structured, conducted virtually using MS Teams post workshop.

### Participants

Eligibility criteria included pre-clinical undergraduate medical students (year 2–3) from Royal College of Surgeons in Ireland (RCSI), Republic of Ireland and Queen’s University Belfast (QUB), UK, before they commence clinical education. The intervention and data collection were conducted online from September 2023 to February 2024; with PROPER workshops held between September and October 2023. Participants were required to complete a 10-minute survey(s), attend a 90-minutes workshop(s) and engage in an online FGD, where applicable. Ethical approval was sought and obtained from the Research Ethics Committee of the RCSI (REC202205007) and QUB (MHLS 22_184) prior to commencement. Clinical trial number: Not applicable.

### Recruitment

Convenient sampling was employed, and recruitment was conducted via email through the Quality Enhancement Office in RCSI. An invitation was emailed to all years 2 and 3 medical students at both institutions (RCSI = 750, QUB = 540) outlining study details, providing a participant information leaflet and a consent form. Participants could select either the *intervention route* (involving pre and post workshop surveys, attendance at one workshop, one post workshop FGD, and a three-month follow up survey) or the *control route* (completing only pre-workshop and follow up surveys with no professionalism education). To encourage retention, participants were rewarded with a certificate of completion and €25 Amazon voucher at the end of data collection.

### PROPER intervention

*PROPER workshops*: four bespoke educational workshops addressing key MP themes: 1. Maintaining confidentiality; 2. Raising concerns and whistle-blowing; 3. Self-care and wellbeing and 4. Exercising cultural sensitivity. Experts from Ireland, UK, Bahrain, and the United Arab Emirates informed the workshop design using educational theories [17]. As presented in Table 1, each workshop was delivered online, joined by participants from RCSI and QUB at the same time, and included a 40 min group scenario analysis. The scenario analysis session employed four scenario of unprofessional behaviour for each workshop theme utilised reflection using Rolfe’s reflection model [18] (Table 1). The scenarios were informed by experts and student panel, with full details provided in our previous publication [17].

**Table 1:** PROPER workshop structure

Stages	Duration	Virtual Online Room	Details
Introduction	5 minutes	Main room (All participants)	Welcome, introductions and overview of workshop objectives
Didactic lecture	10 minutes	Main room (All participants)	Summary of pre-workshop materials and theme
Scenario- analysis	40 minutes	Breakout rooms (4–5 students; 2 facilitators/room) Four scenarios per workshop theme.	Scenario deliberation using Rolfe’s reflective model, problem-solving, and clinical reasoning. Working through four scenarios/ workshop theme
Case-feedback & discussion	25 minutes	Main room (All participants)	Reflecting on hidden curriculum challenges, sharing insights, and discussing lessons learned.
Wrap up	5 minutes	Main room (All participants)	Feedback, conclusion and preview future practice.

#### Quantitative design

The PROPER study survey was adapted from Medisauskaite et al. [19], and validated for relevancy and clarity [20]. The validated survey assesses participants attitude, subjective norms (SN), perceived behaviour control (PBC) and intentions. The survey also collected demographic data including participant’s age, gender, ethnicity and year of study, and was administered online using Survey Monkey^®^.

PROPER intervention impact on PB was assessed by analysing changes within the intervention group from pre-workshop to post-workshop. Change from the pre-workshop to three months follow-up were compared between intervention and control group.

#### Qualitative design

A TPB-based semi-structured guide was developed, piloted, and refined by the research team. Virtual FGDs, audio-recorded with consent, were auto-transcribed, and examined prior to analysis.

### Outcomes

The primary outcomes were the average scores for attitudes (8 items), SN (12 items), PBC (4 items) and intentions (4 items), which were rated on a 7-point scale.

### Data collection

Each participant received a unique ID. One week before the workshops, all consenting participants were sent a pre-workshop survey. Intervention participants received an MS Teams invitation and educational resources related to the workshops. Post-workshop, they completed the post workshop survey and received a FGD invitation. Three months later, all participants received a follow-up survey, following GDPR guidelines [21] .

### Analysis

#### Quantitative analysis

Demographic information was summarised using descriptive statistics. The average score of each TPB construct was calculated at each time point. Sample t-tests were originally planned for analyses, however given the small sample size and statistical power, differences within the intervention group between pre and post intervention were assessed using non-parametric sign test, while comparisons of pre-workshop to three-months follow-up scores between intervention and control groups utilised Mann-Whitney U test. Statistical analyses were performed using Stata v18 and a p-value *≤* 0.05 indicated statistical significance.

#### Qualitative analysis

Thematic analysis followed Braun and Clark’s Six stages approach [22]. Three researchers (AS, SSG and GK) analysed the FGDs using a multi-layered approach, applying a deductive TPB-based coding process. SSG and GK independently coded the first two transcripts before resolve discrepancies with AS. A second inductive analysis followed the same process, ensuring nuances insights during an in-depth analysis.

### Triangulation

Qualitative and quantitative results were triangulated using the Cathkin et al., triangulation method [23]. This methodology allowed for comparison and contrast of the findings to uncover convergence (agreement), complementarity (providing additional insights), or dissonance (contradictions).

### Sample size

For the quantitative arm, power calculations (Cohen’s d = 0.5, 90% power, and a significance level of 0.05) [24] recommended 40 participants per group and adjusted to 60 for attrition and clustering. However, the final sample was smaller than expected, limiting the study’s power to detect small-to-medium effects. For the qualitative arm, a target of 5–8 participants per session was set to ensure interactive discussions [25]. Notably, blinding was not implemented as this was a quasi-experimental study.

## Results

### Participant characteristics

As presented Tables 2, 41 participants took part in the study (Intervention = 31, Control = 10). Participants were recruited from two institutions located in the Republic of Ireland (RCSI: *n* = 27, 65.8%) and UK (QUB: *n* = 14, 34.1%). Equal participation was observed from Years 2 and Year 3 students, predominantly female (*n* = 22, 70.7%) and aged 19–24 years (*n* = 34, 89.9%). Ethnic backgrounds, including Asians (*n* = 24, 58.5%), White (*n* = 12, 29.3%) and Africans (*n* = 5, 12.2%).

**Table 2:** Table of demographics

Category	Intervention group (*N* = 31)	Control group (*N* = 10)	Total participants (*N* = 41)	*p*-value*
Institution *n* (%)				
RCSI	20 (64.5%)	7 (70.0%)	27 (65.8%)	0.99
QUB	11 (35.5%)	3 (30.0%)	14 (34.1%)	
Academic year *n* (%)				
Year 2	18 (58.1%)	5 (50.0%)	23 (56.1%)	0.72
Year 3	13 (41.9%)	5 (50.0%)	18 (43.9%)	
Gender *n* (%)				
Female	22 (71.0%)	7 (70.0%)	29 (70.7%)	0.99
Male	8 (25.8%)	3 (30.0%)	11 (26.8%)	
Non-binary	1 (3.2%)	0 (0.0%)	1 (2.4%)	
Age *n* (%)				
19	7 (22.6%)	3 (30.0%)	10 (24.4%)	0.82
20	11 (35.5%)	3 (30.0%)	14 (34.1%)	
21	7 (22.6%)	3 (30.0%)	10 (24.4%)	
> 21	6 (19.4%)	1 (10.0%)	7 (17.1%)	
Ethnicity *n* (%)				
Asian	20 (64.5%)	4 (40.0%)	24 (58.5%)	0.25
White	7 (22.6%)	5 (50.0%)	12 (29.3%)	
African	4 (12.9%)	1 (10.0%)	5 (12.2%)	

*RCSI *Royal College of Surgeons, South of Ireland, Ireland,* QUB *Queen’s University Belfast, North of Ireland, United Kingdom

^*^Chi-square test used for larger sample sizes and Fisher’s Exact Test when any expected cell count was less than 5

### Quantitative results

In the intervention group, participants were distributed according to preference across four workshops addressing themes of MP: confidentiality (*n* = 11), cultural awareness (*n* = 6), raising concerns (*n* = 6) and selfcare (*n* = 8). The most notable change was observed in PBC for self-care, which significantly improved from pre- to post-workshop (median (IQR): 3.7 (3.2–4.0) to 5.2 (4.9–5.3); *p* < 0.01). For other constructs, improvements were observed but did not reach statistical significance. For example, PBC for cultural awareness increased (5.8 (4.5–6.0) to 6.5 (6.3–6.8); *p* = 0.06), and intentions to raise concerns rose slightly (5.2 (4.7–5.5) to 5.5 (5.0–6.0); *p* = 0.06). Other TPB items showed similar directions of change, except for attitudes to cultural awareness, subjective norms for raising concerns and self-care, and intentions for cultural awareness, where decreases or no improvements were observed (Additional File 1). Other items show positive trends although interpretation is limited by sample size. There was no evidence of a difference from pre-workshop to three-month follow-up when comparing intervention and control groups (Additional File 1).

### Qualitative results

**Fig. 1:**
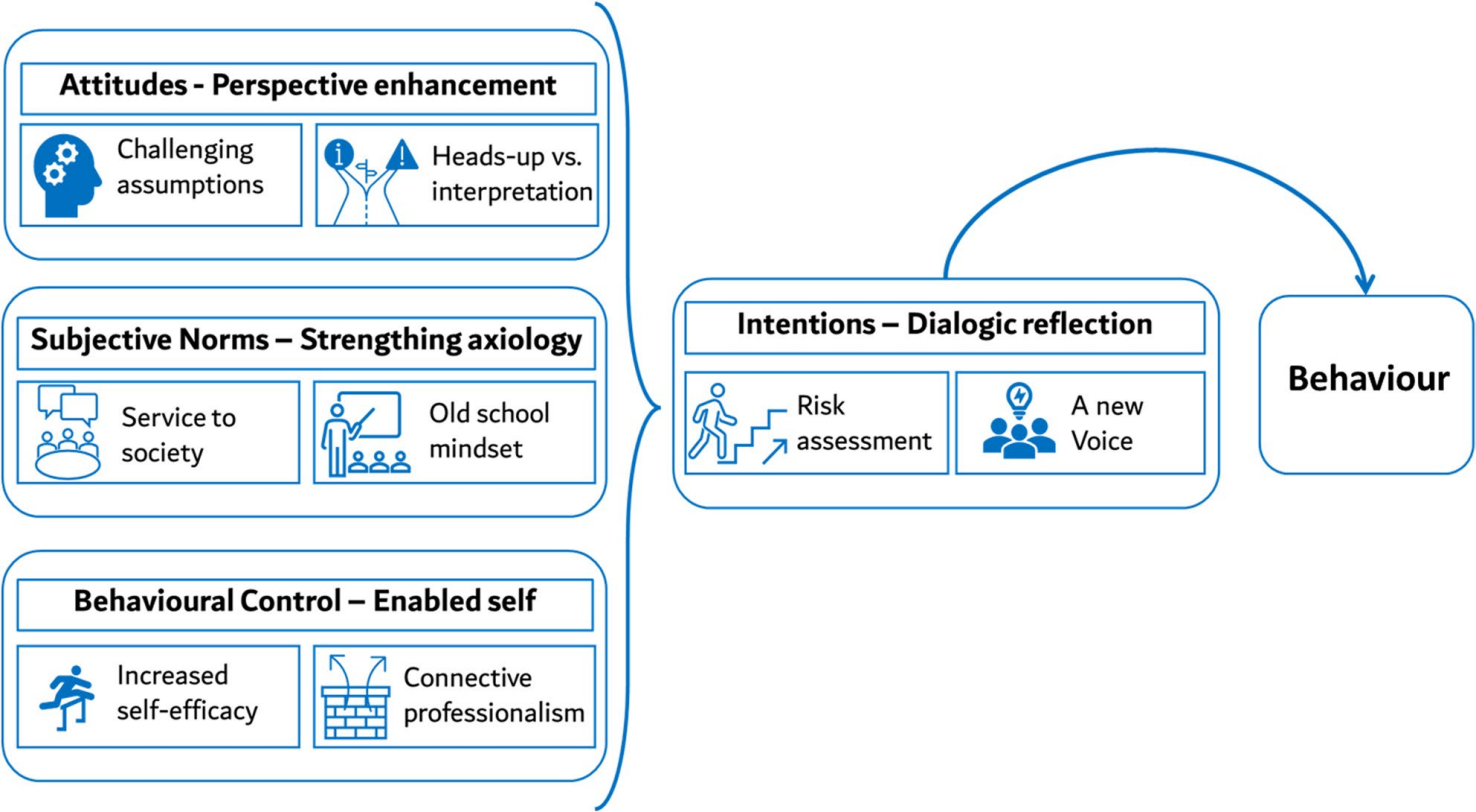
Qualitative sub-themes to behaviour change constructs

Twenty-four participants from the intervention group engaged in 5 online FGD post-workshop, unaware of quantitative results. Each FGD included 4–5 participants and one facilitator and was approximately 60 min in duration. Using the four TPB constructs, data were thematically identified under the four TPB construct, followed detailed exploration of the subthemes, which are mapped in Fig. 1 and elaborated upon in the following text.

#### Attitude: perspective enhancement

Participants reported enhanced perspectives on MP, enabling them to challenge previous assumptions and beliefs. Participants repeatedly conveyed that PROPER intervention prompted them to re-examine preconceptions about MP’s role in students’ lives and in clinical practice. This reflected a contextual understanding of MP that evolves with learning.

*“I think also just being aware of the kind of scenarios where I used to think that professionalism didn’t matter*,* but realizing that it does matter.*,* definitely it’s helped me be a lot more aware of how I act.” Student_23*

This shift suggested a deeper, context-dependent understanding of MP as a phenomenon requiring ongoing reflection.

*“Once you scratch the surface*,* you realized how much there is below… There’s so much to this!” Student_11*.

#### Subjective norms: strengthening axiology

SNs reflected as strengthened axiology, a deepening understanding of values. Participants articulated a strengthened sense of their professional values, enhancing their decision-making abilities in professional contexts.

*“I just feel like as long as I have my views and my views are in charge and I still believe in them*,* I’ll be able to be more professional.”* Student_04

They emphasised the importance of having clear professional beliefs for building patient trust.

*“If hidden curriculum is shared with patients and they understand that we are working on it and trying to improve it*,* it will make them feel safer and build more trust in the medical professional in a way.”* Student_22

However, some concerns were raised about how certain peers’ attitudes towards professionalism could undermine their axiological belief growth or their practice development.

*“Older generations who are not open minded to changes in how the medical professional is perceived or people think professionalism is a waste of time…and some professors who really don’t agree with professionalism.” Student_03*

#### Perceived behaviour control: enabling self

From PBC analysis emerged ‘enabled self’ which was characterised by increased confidence in handling future professional dilemmas. Many participants felt reassured about their knowledge and abilities post-workshop. Small group discussions further solidified these understandings.

*“You’re getting reassurance that you know what’s right to do and that you can put it into practice because you’ve thought through those experiences…. And I think that I was aware of what to do before*,* but I think the workshop reiterated that and consolidated my like knowledge.”* Student_20

However, some participants expressed the need for continued reinforcement of the newfound self-efficacy.

“*I feel a bit more confident than prior to the study*,* but I’m still not 100% confident in myself and my ability… But hopefully I guess by the time that would be 100%.”* Student_16

Participants feared medical hierarchical practices could undermine confidence, influencing their PB practices.

*“I could look at the guidelines of the PROPER study and try to do something*,* but if my superior is not OK with that*,* even if I want to do the change and I’m unable to do the change.”* Student_22

#### Intentions: dialogic reflection

Intentions manifested as dialogic reflections, demonstrating improved risk assessment capabilities and ability to reflect critically on their actions.

*“I think I’ve already applied at least some of them while being in clinical practice and kind of noticed the bias and the differences in my thinking that I’ve noticed after doing one of the workshops.”* Student_14

Participants articulated their improved confidence in rationalising the right approaches, and consider translating acquired theoretical insights to future professional practices.

*“Overall*,* my confidence has increased*,* but I think it’ll increase as I actually have to deal with real life situations and I take the initiative to use them to use the PROPER study hidden curriculum.”* Student_23

Dialogic reflections also revealed that participants felt empowered to express their views and challenge the existing hierarchy constructively.

*“I think I’m more vocalizing now. I’m trying to tell them [seniors] in the politest way and raise concerns when needed even in work or anywhere out of work.…. Before I used to not say anything.”* Student_23

### Triangulation

Quantitative and qualitative results were triangulated using O’Cathain *et al.’s framework*, summarising themes in a table to compare results interpretable for convergence, complementarily and discrepancies. This method facilitated deeper understanding and interpretation of the combined results (Table 3). In short, attitudes quantitative data showed no significant change, while qualitative accounts suggested enhanced perspectives; this reflects dissonance likely due to limitations of survey measures in capturing nuanced shifts. For subjective norms, the absence of quantitative improvement contrasted with qualitative evidence of strengthened values and recognition of barriers, indicating complementarity where the qualitative strand added contextual depth beyond numerical outcomes. In perceived behavioural control, both strands pointed to increased confidence and self-care, demonstrating clear convergence, though qualitative insights also highlighted hierarchical threats to these improvements. For intentions, quantitative improvements aligned with qualitative reflections on dialogic reasoning, again showing convergence. Finally, in behaviour change sustainability, quantitative results showed no sustainable impact, whereas qualitative findings emphasized the need for iterative reinforcement, marking complementarity and underscoring measurement limitations in reporting long-term changes.

**Table 3:** Triangulation

Theme	Quantitative	Qualitative
Attitude	No significant improvement in attitudes towards raising concerns	Enhanced perspectives and understanding of MP attitudes
Subjective norms	No significant improvement in subjective norms across MP theme	Strengthened values and beliefs within social structures, alongside barriers and facilitators, were recorded.
PBC	Significant improvement in PBC related to selfcare and some evidence of improvement towards cultural awareness	Increased confidence in engaging in professional practices, though hierarchical practices were seen as a threat to the confidence and newly acquired MP understandings.
Intentions	Some evidence of improvement in intentions to raising concerns	Dialogic reflections showed improved ability to assess benefits and threats of actions in professionalism in practice.
Behaviour change Sustainability	No significant sustained impact on behaviour change over time compared to control group	Participants emphasized the need for iterative efforts to maintain self-efficacy and confidence.

*MP *Medical professionalism,* PBC *Perceived behaviour control

## Discussion

MP education is an essential component of undergraduate teaching. PROPER intervention resulted in some evidence of improvements in a number of behaviour constructs towards raising concerns, self-care and cultural awareness. Qualitative findings revealed four emergent themes that provided further details to the improvement journey in PB, these included: *perspective enhancement*, *strengthening axiology*, *enabling self*, and *dialogic reflection*. These findings emphasize the impact of PROPER on personal growth and readiness of medical students to act professionally. However, contextual barriers (e.g., hierarchy), were reported to have hindered the students’ significant improvement and the perceived readiness for long-term translation of intentions into behaviour changes. Moreover, PROPER study demonstrates the feasibility of delivering professionalism-focused education online across two institutions.

While attitudes scores showed no statistical improvements towards raising concerns but rather a positive trend using the sign test, qualitative evidence revealed participants’ expanded understanding of MP and its clinical importance. Perspective enhancement evidenced a revaluation of MP’s significance in clinical practice, mirroring emerging research that highlights the importance of cognitive understanding in influencing professional behaviour and potentially shaping identity [26]. This shift reflects a deeper cognitive evolution beyond surface-level understanding. These results resonate with findings from previous systematic reviews emphasising the benefits of structural educational frameworks and reflective practices [9, 27]. Johnsen et al., [28] also suggested that similar interventions can ease inner conflict in raising concerns, emphasizing the complexity of attitudes and the need for targeted strategies to ensure patient safety. PROPER interventions have contributed to enabling undergraduate students’ ability to internalize professional values by emphasizing professionalism learning. This targeted approach fosters deeper reflection, ethical awareness, and behavioural integration of core professional standards. While quantitative analysis showed no significant changes in SNs, qualitative data indicated a notable development in participants’ professional values, particularly through external validation from peers and patients. This observation aligns with the intervention’s design, which is grounded in social constructivist theory [17]. This approach emphasises learning within the zone of proximal development, where social interactions, shared experiences and cultural/social context facilitate the assimilation of sociocultural behaviours and transformation of learner identities [29–31]. Additionally, participants recalibrated their moral compasses, reflecting strengthened axiology, where learners construct understanding through social engagement [32]. However, the lack of quantitative change of SNs may reflect measurement limitations, which might not capture the nuanced shifts more observable through qualitative methods. Literature suggests that SNs are context-dependent, and challenging to quantify effectively [33].

PROPER study results reveal these challenges in form of traditional professionalism perspectives which students reported to be held by senior healthcare professionals, which can act as a barrier to student’s PB developments. Medical students often model their behaviours on their supervisors’, adopting their values and behaviours, despite undergoing attitudinal shifts [34, 35]. This can suppress the evolution of professional identity aligned with modern professional standards [36, 37]. This highlights the need to promote evidence-based PBs that reflect current ethical standards, inclusivity, and patient-centred care rather than hierarchical norms. PROPER intervention aims to integrate these values into educational curricula, allowing students to internalize PBs consistent with evolving societal expectations [13, 38]. This alignment creates a supportive learning environment where students can challenge traditional perceptions, prioritise collaboration and ensure patient safety.

Quantitative findings revealed statistically significant and potentially meaningful improvements in PBC, particularly in self-care and cultural awareness. These results were reinforced by qualitative insights, where participants expressed increased potential confidence in applying the professional behaviours acquired during the clinical stage of education and practice. The PROPER interventions validated existing knowledge taught on professionalism topics embedded within other courses within RCSI participants, strengthening participants’ belief in their professional abilities. However, confidence was perceived as context-dependent, potentially threatened by hierarchical environments that undermine PBC. This suggests that increased PBC may not drive behaviour change if barriers are present [39]. These findings suggest the necessity for parallel interventions to mitigate hierarchical norms in clinical settings, fostering an environment where professionals can express their behaviours without structural limitations. This observation is consistent with the TPB framework, which suggests that increased PBC does not always lead to behaviour change when situational constraints exist [40]. The literature identifies PBC as a challenging construct due to its complexity [33, 39]. Research on PBC within medical education remains scarce and while some interventions (i.e., workshops) aimed at modifying PBC have inconsistent results, comprehensive strategies remain limited [41–43]. The novelty of our PROPER intervention lies in its design, incorporating collective reflections using Rolfe’s model, and expert guidance to address real-world scenarios [18]. This approach fostered self-efficacy, psychological safety and PB. Aligning with Mann et al., our findings on reflective processes deepens professional awareness, examine assumptions and readiness for professionalism in practice [44]. Additionally, large group discussions in PROPER workshops helped reinforce new insights, challenges, and strengthen PBC among participants.

In a similar manner, potential improvements in intention to raising concerns scores were reinforced by qualitative insights, revealing participants’ engagement in dialogic reflections. The correlation between intentions and behaviour within our framework, is well-documented; for instance, Henderman et al., highlighted TPB-based intentions reliably predict behaviour change [45]. PROPER intervention have the potential to foster meaningful introspection among medical students, with short-term gains in knowledge, confidence, and self-reflection observed. However, the lack of statistically significant effects at the 3-month follow-up highlights several challenges. First, limitations in sample size and statistical power may have reduced the ability to detect sustained effects at follow up time. Second, while features of the intervention frequency and duration (one time workshop) have improved some constructs at short-term, however may have been insufficient to consolidate behavioural change over time. Third, contextual and cultural barriers, particularly hierarchical norms, organisational culture, and fears of repercussions for raising concerns, further impeded the translation of professionalism learning into practice [46–48]. Addressing these methodological and structural factors alongside professionalism education is crucial to create an environment where professional behaviours can be enacted without fear of negative consequences, thereby supporting the sustainability of professionalism learning.

### Strengths and limitations

PROPER study is a theory-based project, designed to enhance MP education for culturally diverse students from two different institutions in Northern and Southern Ireland, employing mixed method, quasi-experimental design. Experts and students co-developed PROPER intervention to ensure contextual relevance, and validated questionnaires were used to accurately assess behaviour change. The virtual format improved accessibility and engagement for geographically dispersed learners, demonstrating the feasibility of cross-institutional and cross-border professionalism teaching. Beyond this context, the study offers transferable insights by showing curated workshop and structured scenario analysis, dialogic reflection, and value‑based deliberation can support professionalism development across diverse settings. Importantly, the findings show that professionalism cannot be developed through value‑based teaching alone; it requires deliberate alignment between educational strategies, the clinical environments and institutional cultures which play a decisive role in either enabling or constraining professional behaviour. As such, the PROPER intervention highlights not only the value of structured, theory-informed teaching, but also the need for parallel efforts to shape clinical environments that consistently reinforce rather than undermine the professional behaviour promoted in formal curricula.

The study faced several limitations. First, recruitment challenges led to a limited quantitative sample size, which constrained statistical power and precluded advanced analyses such as sustainability comparisons and structural equation modelling. On reflection, embedding the study within the formal curriculum with optional consent to participate in research may have enhanced both recruitment and retention, but was not considered logistically possible across two separate institutions. Second, the self-selection of participants into intervention and control groups may have introduced selection bias, as differences in motivation or baseline characteristics could have influenced outcomes independent of the intervention. Third, the lack of a control group at immediate post-intervention (Time 2) for comparison. Forth, issues related to data sufficiency and the potential loss of subthemes were noted in both the quantitative and qualitative analyses. While these constraints are acknowledged, the integration of qualitative and quantitative data through methodological triangulation was intentionally employed to enhance the credibility and depth of the results. Triangulation does not eliminate the limitations associated with sample size but provides complementary insights that strengthen the internal validity and contextual understanding of the intervention’s effects. However, the study provides meaningful insights into the intervention’s impact within a naturally occurring, self-directed educational context. Furthermore, the qualitative findings offer rich, contextualized perspectives that may inform a blueprint for future MP educational interventions at different institutions and geographics. Future research with larger and more diverse samples is warranted to confirm and extend these findings.

## Conclusion

PROPER study offers a promising blueprint for online professionalism education, combining structured learning and reflection to foster collaboration and cultural competence. While improvements in PB were noted, hierarchical norms and limited clinical experience may have constrained long-term change. Future interventions should target barriers via faculty development and experiential strategies to translate and foster behaviour change in clinical practice. Additionally, future studies are recommended to employ random allocation or matched designs in the recruitment to reduce bias and strengthen causal inference.

## Supplementary Information

Supplementary Material 1.

## Data Availability

The datasets used and/or analysed during the current study are available from the corresponding author on reasonable request.

## Abbreviations

PROPER: Professionalism in Partnership for Education Research
MP: Medical professionalism
PB: Professional Behaviour
TPB: Theory of Planned Behaviour
SN: Subjective norms
PBC: Perceived Behaviour Control
