# Supplementary material for: PROfessionalism in Partnership for Education Research (PROPER) study: a novel online initiative approach to professionalism education
Source: BMC Med Educ. 2026 Apr 29;26:913. doi: 10.1186/s12909-026-08908-2 (PMC13234974; doi:10.1186/s12909-026-08908-2)
Supplement: Supplementary file 1 — Supplementary Material 1. [file 12909_2026_8908_MOESM1_ESM.docx]

Additional File 1: Scores Comparison for Intervention and control Groups across Behaviour Constructs and Themes, with p-values

|  |  | **Intervention** | | | | **Control** | | |
| --- | --- | --- | --- | --- | --- | --- | --- | --- |
| **Measure** | **Theme** | **Time 1 Median (IQR)** | **Time 2 Median**  **(IQR)** | **Sign Test p-value Time 2 - Time 1** | **Time 3 Median (IQR)** | **Time 1 Median (IQR)** | **Time 3 Median (IQR)** | **Intervention v Control Time 3 – Time 1** |
| **Attitude** | Confidentiality | 6.0 (6.0–6.4) | 6.3 (6.0–6.6) | 0.453 | 5.8  (5.7–6.2) | 6.2  (5.8–6.2) | 6 (5.5–6.5) | 0.9219 |
|  | Cultural Awareness | 5.7 (5.3–6.1) | 5.4 (5.3–6.4) | 0.999 | 5 (4.7–5.8) | 5.6 (4.2–6.7) | 4.6 (2–5.4) | 0.3042 |
|  | Raising concerns | 5.1 (4.8–6.4) | 6.3 (6.2–6.4) | 0.219 | 5.8 (5.5–6) | 5.9 (5–6.2) | 4.8 (3.9–5.6) | 0.1264 |
|  | Self-care | 5.4 (5.1–5.5) | 5.5 (5.1–5.9) | 0.453 | 5.8 (5.3–6.1) | 5.1 (4.3–5.4) | 4.3 (2–5.5) | 0.1538 |
| **Subjective norms** | Confidentiality | 4.8 (3.3–5.5) | 5.3 (3.0–5.6) | 0.999 | 4.2 (2.6–5.9) | 4.1 (2.0–4.8) | 3.6 (2.4–5.6) | 0.9223 |
|  | Cultural Awareness | 4.5 (3.7–5.5) | 5.1 (4.7–5.9) | 0.688 | 4.3 (4.2–5.1) | 5 (4.08–5.5) | 4.8 (4.4–5.5) | 0.7150 |
|  | Raising concerns | 4.8 (4.2–5.2) | 4.6 (3.9–5.7) | 0.999 | 4.7 (3.4–5.9) | 3.5 (3.08–4.3) | 4.8 (4–5.6) | 0.1074 |
|  | Self-care | 4.0 (3.8–4.2) | 4.0 (3.8–4.5) | 0.727 | 4.3 (4.08–4.5) | 3.7 (3.08–4.3) | 4.4 (4–5.3) | 0.4233 |
| **Perceived behaviour control** | Confidentiality | 4.8 (4.4–5.0) | 5.4 (4.2–5.8) | 0.999 | 4 (3.7–4.7) | 4.2 (3.7–4.5) | 4.2 (3.7–5.2) | 0.1850 |
|  | Cultural Awareness | 5.8 (4.5–6.0) | 6.5 (6.3–6.8) | 0.063 | 5.6 (5.3–7) | 6 (5.5–6.5) | 5.2 (5–7) | 0.7144 |
|  | Raising concerns | 4.8 (3.8–5.6) | 6.0 (5.4–6.4) | 0.219 | 4.2 (4–4.7) | 4.8 (4.1–5.7) | 4.5 (4–4.8) | 0.2312 |
|  | Self-care | 3.7 (3.2–4.0) | 5.2 (4.9–5.3) | **0.008** | 4.8 (4.7–5.2) | 4.5 (4–5) | 4.5 (4–6.5) | 0.1720 |
| **Intentions** | Confidentiality | 4.7 (3.7–5.3) | 5.0 (4.7–6.0) | 0.348 | 4.3 (3.6–4.6) | 4.6 (4–5) | 4.6 (4–5.3) | 0.9218 |
|  | Cultural Awareness | 6.6 (5.8–7.0) | 6.5 (6.5–7.0) | 0.999 | 6.5 (6–7) | 6 (4.1–6.3) | 5.1 (2.1–6.2) | 0.6059 |
|  | Raising concerns | 5.2 (4.7–5.5) | 5.5 (5.0–6.0) | 0.063 | 5.4 (4.08–5.8) | 4.8 (4.4–4.9) | 4.5 (3.7–5.08) | 0.3065 |
|  | Self-care | 6.0 (5.5–6.3) | 6.3 (5.6–6.5) | 0.219 | 6.1 (6–6.5) | 4.3 (4–5.2) | 4.1 (2–5.3) | 0.1189 |

***IQR: Interquartile Range***
